# Climate change effects on Black Sigatoka disease of banana

**DOI:** 10.1098/rstb.2018.0269

**Published:** 2019-05-06

**Authors:** Daniel P. Bebber

**Affiliations:** Department of Biosciences, University of Exeter, EX4 4QD Exeter, UK

**Keywords:** *Musa*, Black Leaf Streak Disease, *Pseudocercospora fijiensis*, *Mycosphaerella fijiensis*, epidemiology, invasive species

## Abstract

Climate change has significantly altered species distributions in the wild and has the potential to affect the interactions between pests and diseases and their human, animal and plant hosts. While several studies have projected changes in disease distributions in the future, responses to historical climate change are poorly understood. Such analyses are required to dissect the relative contributions of climate change, host availability and dispersal to the emergence of pests and diseases. Here, we model the influence of climate change on the most damaging disease of a major tropical food plant, Black Sigatoka disease of banana. Black Sigatoka emerged from Asia in the late twentieth Century and has recently completed its invasion of Latin American and Caribbean banana-growing areas. We parametrize an infection model with published experimental data and drive the model with hourly microclimate data from a global climate reanalysis dataset. We define infection risk as the sum of the number of modelled hourly spore cohorts that infect a leaf over a time interval. The model shows that infection risk has increased by a median of 44.2% across banana-growing areas of Latin America and the Caribbean since the 1960s, due to increasing canopy wetness and improving temperature conditions for the pathogen. Thus, while increasing banana production and global trade have probably facilitated Black Sigatoka establishment and spread, climate change has made the region increasingly conducive for plant infection.

This article is part of the theme issue ‘Modelling infectious disease outbreaks in humans, animals and plants: approaches and important themes’. This issue is linked with the subsequent theme issue ‘Modelling infectious disease outbreaks in humans, animals and plants: epidemic forecasting and control’.

## 1. Introduction

Over the past century, human activities have dramatically altered the Earth's atmospheric composition, with significant consequences for the planet's climate, biogeochemistry, ecosystems and societies [1]. Biological processes are strongly influenced by temperature and water availability, and the influence of recent anthropogenic climate change on ecosystems is well documented, with hundreds of examples of latitudinal migrations and changing phenologies in response to warming [2]. Like other organisms, pests, parasites and diseases are influenced by abiotic conditions; therefore, climate change is expected to alter the distribution and impact of these organisms on their human, animal and plant hosts [3–7].

Crop pests and pathogens are spreading rapidly around the world [8], and latitudinal shifts in the distributions of these organisms since the 1960s [9] are largely in line with expectations of climate change [10]. These damaging organisms can have particularly severe economic consequences in the developing world, given the greater dependence of developing countries' economies on agriculture, and the relative lack of resources and technological capacity to control crop disease [11]. Among the most important tropical crops, as both a dietary staple and an exported cash crop, is the banana (*Musa* spp.). Bananas and plantains are the seventh most important crop by production in the developing world, with India, China and Brazil being the most important producers [12]. It is therefore of great concern that pests and diseases of bananas have been among the most rapidly spreading of all crops in recent years [3]. The re-emergence of Fusarium Wilt (*Fusarium oxysporum* f. sp. *cubense*), also known as Panama Disease, in the form of Tropical Race 4 (TR4) from Southeast Asia, is of particular concern to the export industries of Latin America and the Caribbean, because planted cultivars of Cavendish bananas are highly susceptible to the disease [13].

While the focus on control of TR4 is justified given potential production impacts and economic consequences, Fusarium Wilt is not currently the most important disease in banana production globally. Another fungal disease, known as Black Sigatoka or Black Leaf Streak Disease, has recently emerged from Asia and now causes the greatest yield losses in banana plantations globally [14]. Black Sigatoka is caused by the Ascomycete fungus *Pseudocercospora fijiensis* (formerly *Mycosphaerella fijiensis*). *P. fijiensis* spreads via aerial spores, infecting banana leaves via the stomata and causing characteristic streaked lesions and cell death when fungal toxins are exposed to light [15]. The disease is virulent against a wide range of banana genotypes, and infected plant yields are reduced by up to 80% if untreated [14].

Establishment of an emerging plant disease in new territories requires the presence of the host and a suitable climate [3]. Thus, while dispersal via international trade [16] or via spores drifting on the wind [17] may be the means of arrival, climate change may alter abiotic conditions that make the establishment of a disease more or less likely. Studies have attempted to model potential future changes in plant pest and pathogen distributions and impacts [18–21], but relatively few have considered how historical climate change has altered pest and disease burdens on agriculture. By applying models that estimate disease risk over time, we can infer whether conditions have become more or less conducive to particular plant diseases and thus determine any ‘fingerprint’ of climate change. Here, we develop epidemiological and statistical models for Black Sigatoka using published data, and drive these models using historical climatic data for the banana-growing regions of Latin America and the Caribbean. We test the hypothesis that climate change over the past 60 years has increased the risk of Black Sigatoka outbreaks, and discuss how future climate change might influence this important disease of bananas.

## 2. Material and methods

The life cycle of *P. fijiensis* is strongly determined by weather and microclimate [22]. Ascospores infect leaves through the stomata, with infection producing necrotic lesions that eventually develop conidia that can lead to secondary infections, again via the stomata. Conidia are not thought to be important sources of infection, because *P. fijiensis* forms relatively few of them in comparison with ascospores. Ascospores are dispersed over long distances by wind [23], while conidia form readily under wet or humid conditions and are dispersed by rain-splash. Like many foliar fungal pathogens, *P. fijiensis* spores require a wet leaf surface or very high relative humidity (RH) to germinate and infect the leaf, and the rate of germination and infection during wet or humid periods is governed by temperature [24].

Several experimental studies have investigated the dependence of spore germination, infection and Black Sigatoka disease development on microclimate. Uchôa *et al*. [24,25] measured infection and disease development rates in relation to temperature and leaf wetness duration (LWD). We abstracted area under the disease progress curve (AUDPC) values under a variety of temperatures and LWD from figure 2, p. 83 in [25] to parametrize a new disease development model. Given that RH was reduced to around 55% after controlled periods of leaf wetness in that study, we assume that all germination and penetration occurred during the wet periods. We interpreted the AUDPC as an outcome of infection level driven by the germination and penetration of spores, the rates of which are dependent upon temperature and LWD. In reality, the AUDPC is monotonically but not necessarily linearly related to the number of infections, though we assume linearity in the absence of further data. We did not model other aspects of disease development such as the latent period (but see electronic supplementary material).

We treated infection as a probabilistic survival process of spores transitioning to infections, which proceeds over time *t* during wet periods and has a Weibull hazard function *H* dependent upon temperature *T* (equations (2.1) and (2.2)). The temperature-dependent rate *r* is determined by the cardinal temperatures, namely the minimum (*T*_min_), optimum (*T*_opt_) and maximum (*T*_max_) (equation (2.3)). Full details of the model are provided in ref. [26] and in electronic supplementary material. We estimated the following model parameters by simulated annealing optimization [27]: *T*_min_, *T*_opt_, *T*_max_, the scale factor of the Weibull hazard function *α*, the shape parameter of the Weibull hazard function *γ* and a scaling factor *β* such that *βF*(*t*) = AUDPC(*t*) where *F*(*t*) is the fraction of a cohort of spores that have germinated by time *t*.

2.1 $$F(t,T)=1-e^{-H(t,T)}.$$

2.2 $$H(t,T)=r(T){\left(\frac{t}{\alpha }\right)}^{\gamma }.$$

2.3 $$\mathrm{and}\;r(T)=\left(\frac{T_{\max}-T}{T_{\max}-T_{\mathrm{opt}}}\right){\left(\frac{T-T_{\min}}{T_{\mathrm{opt}}-T_{\min}}\right)}^{T_{\mathrm{opt}}-T_{\min}/T_{\max}-T_{\mathrm{opt}}}.$$

As previously [26,28], we employed the Japanese Meteorological Agency 55-Year reanalysis (JRA55) to model the role of weather on infection [29]. We obtained 3-hourly JRA55 estimates at 0.5625° × 0.5625° resolution (approx. 60 km grid) for plant canopy temperature, canopy surface water and RH at 2 m above the ground, from the US National Center for Atmospheric Research (NCAR) [30], for the period 1958–2017 inclusive. Our region of interest was tropical Latin America and the Caribbean, 32.625° W to 109.6875° W and 23.0625° S to 23.0625° N, a grid of 82 rows and 137 columns containing 3907 land pixels. Of these, we selected 830 pixels estimated to contain greater than 0.1% banana-growing area (of the pixel area) in the SPAM dataset of global crop production [31] for further analysis. In Latin America, bananas tend to be grown at low elevations, reducing the within-pixel variability in temperature due to topography, so we did not correct for the elevational lapse rate (see electronic supplementary material).

For each pixel, we linearly interpolated the 3-hourly micro-climate data to hourly, then modelled cumulative infection probabilities for hourly cohorts of spores, with rates determined by our temperature response function. The hazard function was calculated piecewise by the temperature in each hour of each wet period. We assumed that ascospores had identical temperature requirements to conidia, as ascospore data are unavailable. Cohorts were assumed not to accumulate on leaves during dry periods, i.e. only one cohort began to germinate in each hour under conditions of leaf wetness or with RH greater than or equal to 98%. Cohorts were assumed to stop germinating and die under drier conditions. The cumulative number of infections was summed over all preceding cohorts for each hour to give *infection risk*, our metric for disease pressure in relation to weather. These were summed to give annual infection risk in each pixel.

Further details of data and analyses are given in the electronic supplementary material, including details of the infection risk model and its parametrization (with computer code examples), additional data sources, details of the JRA55 dataset and consideration of sub-pixel variation in microclimate estimates, the geographical distribution of banana production and an analysis of the effects of weather on the disease incubation and latent periods.

## 3. Results

**Figure 1.**
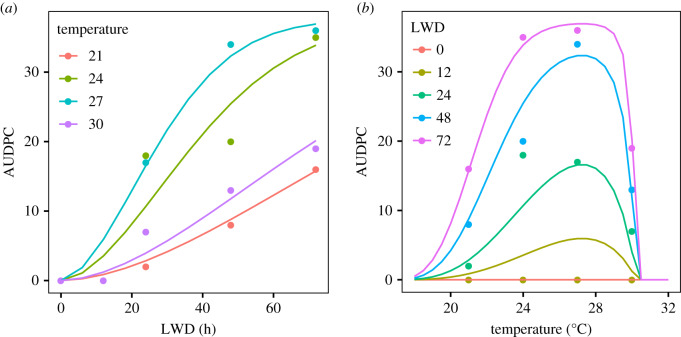
Temperature and LWD response functions for AUDPC. Points show data from [25], coloured by (*a*) temperature and (*b*) LWD. Lines show modelled responses, fitted by optimization of a temperature-dependent Weibull survival function, scaled to the units of AUDPC. The cardinal temperatures are *T*_min_ = 16.6, *T*_opt_ = 27.2 and *T*_max_ = 30.3°C. The Weibull parameters are *α* = 32.6, *γ* = 1.76, and the scaling parameter *β* = 37.6.

**Figure 2.**
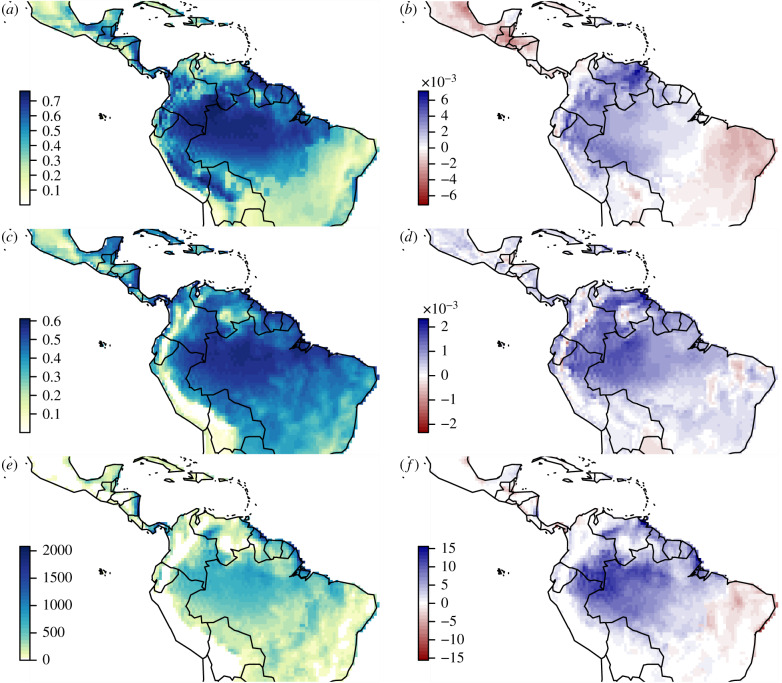
Climate and infection risk in Latin America and the Caribbean, 1958–2017. (*a*) Mean fraction of time during which the canopy was wet enough for *P. fijiensis* infection to occur, i.e. RH greater than or equal to 98% or canopy surface moisture greater than 0. (*b*) Linear annual trend in canopy wetness fraction. (*c*) Mean temperature-dependent rate for *P. fijiensis* infection. (*d*) Linear annual trend in temperature-dependent rate. (*e*) Mean annual infection events derived from infection risk model. (*f*) Linear annual trend in mean annual infection events. Results for the entire region are shown, not only banana-growing areas. Trends should be multiplied by 60 to estimate mean change over the study period.

The best-fitting model parameters for the Uchôa *et al*. [24,25] disease development data were *T*_min_ = 16.6, *T*_opt_ = 27.2 and *T*_max_ = 30.3°C, *α* = 32.6, *γ* = 1.76, and scaling parameter *β* = 37.6 (figure 1). Thus, at *T*_opt_, a cohort of spores would reach 50% of maximum infection after around 26 h of moist conditions, and 98% of maximum infection after around 72 h of moist conditions. Considering the entire Latin American and Caribbean land surface over the period 1958–2017, suitably moist conditions (RH greater than or equal to 98% or liquid water present) were most frequent in the western Amazon basin of Brazil and Colombia, the Andean regions of Bolivia, Peru, Ecuador and Colombia, and southern Panama, while dry conditions occurred in southern Brazil, Mexico, Cuba, coastal Venezuela, northern Colombia (figure 2*a*). Over the study period, the central area comprising western Brazil, Venezuela, southern and central Colombia, much of Ecuador and Peru, and the Dominican Republic became wetter, while Mexico, Central America, Cuba and southern Brazil became drier (figure 2*b*). The canopy temperature suitability for Black Sigatoka infection, as defined by the beta function *r*(*T*), was greatest on average in the Amazon basin, Panama and eastern Nicaragua, and lowest in Mexico and high-elevation regions of the Andes and the Guiana Shield (figure 2*c*). The canopy temperature suitability for infection increased over much of Latin America and the Caribbean, particularly in the Amazon basin, coastal Ecuador and Dominican Republic (figure 2*d*).

Model simulations for infection in hourly cohorts of spores from 00.00 on 1 January 1958 to 23.00 on 31 December 2017 indicated the greatest mean infection risk in eastern Nicaragua, Panama and coastal Guyana (figure 2*e*), with up to 2000 cohorts of spores infecting per year. In context, the model would allow for up to 8760 cohorts to infect per year (this being the number of hours per year). Potential infection risk increased most rapidly across the Amazon basin, and parts of Nicaragua, Ecuador, Guyana, Suriname, French Guiana and the Dominican Republic, and decreased in southern Brazil, much of Central America, and the north coast of Colombia (figure 2*f*). Considering only the banana-growing pixels with greater than 0.1% banana-growing area per pixel (approx. 20% of the region), the median annual average infection number was 234, with a median trend of +0.32 (table 1). Modelled infection risk per pixel in banana-growing areas increased by a median of 44.2% (interquartile range −2.7–95.2%) over that period (table 1). For the top 10 banana-producing nations, Panama had the highest mean annual infection score, followed by Brazil and Colombia, while Brazil had the most rapid increase in infection risk in banana-growing regions (table 1; electronic supplementary material, figure S7). For the entire region, the infection risk trend was somewhat more strongly related to trends in annual temperature-dependent rate (correlation 0.80) than trends in the fraction of time the canopy was wet (correlation 0.71).

**Table 1.** Black Sigatoka disease pressure for banana-growing areas. Summaries are for the top 10 banana-producing countries, and the entire region. Pixels gives the number of pixels in the analysis, containing greater than 0.1% banana-growing area according to the SPAM dataset. Mean and trend give the median and interquartile ranges of mean and trend in *P. fijiensis* modelled annual infection intensity (see main text for details). Change gives the median and interquartile ranges of the relative change in annual infection intensity between the 1960s and the 2000s.

country	pixels	mean	trend	change (%)
Brazil	533	331 (150, 564)	+1.66 (−0.28, +5.38)	39.5 (−8.5, +91.0)
Colombia	79	89 (12, 271)	+0.18 (+0.01, +1.00)	62.2 (29.1, 159.4)
Costa Rica	6	109 (42, 206)	+0.57 (+0.22, +0.93)	88.5 (75.4, 91.4)
Dominican Republic	12	71 (38, 112)	+0.61 (+0.36, +0.98)	40.4 (34.1, 51.7)
Ecuador	42	84 (3, 241)	+0.54 (+0.01, +2.05)	95.0 (66.2, 246.5)
Guatemala	14	43 (7, 144)	−0.31 (−1.20, −0.08)	−40.7 (−57.9, −31.2)
Honduras	21	40 (21, 57)	−0.34 (−0.59, −0.08)	−54.6 (−60.7, −26.7)
Mexico	51	88 (19, 241)	−0.01 (−0.44, +0.22)	39.9 (21.8, 71.4)
Panama	15	785 (559, 1199)	−0.52 (−3.05, 0.77)	2.8 (−3.6, 15.5)
Venezuela	29	99 (50, 175)	+0.59 (+0.10, +1.33)	173.8 (71.8, 238.8)
Region	830	234 (91, 462)	+0.32 (−0.23, +3.63)	44.2 (−2.7, 95.2)

## 4. Discussion

Our analysis shows that Black Sigatoka infection risk has increased significantly across the banana-growing regions of Latin America and the Caribbean, increasing by a median of 44.2% per pixel from the 1960s to the 2010s. This increase in risk was caused by climate change that improved the temperature conditions for spore germination and growth and made crop canopies wetter. In some parts of Mexico and Central America, a drying trend has reduced infection risk. Black Sigatoka was first reported from Honduras in 1972 [32], spreading throughout the region to reach Brazil in 1998 [33] and the Caribbean islands of Martinique, St Lucia and St Vincent and the Grenadines in the late 2000s [34]. The disease now occurs as far north as Florida. While *P. fijiensis* is likely to have been introduced into Honduras on plants imported from Asia for breeding research [35], our models indicate that climate change of the past 60 years has exacerbated the impact of this pathogen.

The spread of Black Sigatoka across Latin America and the Caribbean provides an example of the Biotic–Abiotic–Migration (BAM) framework of Sobéron and colleagues [36], in which the observed distribution of a species is the intersection of biologically and climatically suitable regions which have been reached by a species. The area under banana cultivation has increased over time, the climate has improved and growing international trade and transport have made these suitable areas accessible to the pathogen. While there have been many projections of future changes in plant disease distributions [18–21], investigations of historical changes in plant pathogen distributions and impacts due to climate change are rare [3], and this is also the case with human infectious diseases, where analysis of empirical relations between climatic variables and disease is not often followed by modelling the consequences of changing climate on disease [5]. Given that we have more certainty about historical climate change and disease incidence than we do about the future, further investigations of the consequences of twentieth Century changes may help us to disentangle the relative importance of abiotic, biotic and migratory factors on emerging diseases.

We employed a ‘forward modelling’ approach [37] in which experimentally determined physiological responses are used to estimate infection risk given appropriate weather data [38]. A potential weakness of this approach is that the behaviour of an organism in the laboratory may differ from that in the field, where numerous other environmental and biological factors may be at play [39]. For example, a study of the wheat pathogens *Phaeosphaeria nodorum* and *Zymoseptoria tritici* in the UK revealed air pollution as the main driver of their relative abundance [40], invalidating any model based purely on climate responses for periods over which air quality has changed significantly. Our model considered only the spore germination and infection processes, which decades of research have found are controlled primarily by temperature and water availability [14,22,41–43]. Hence, it is unlikely that we have omitted other important drivers. Nevertheless, detailed observational studies of disease incidence over a wide geographical area would be valuable for validation of our model predictions, though sufficiently detailed historical records do not exist. We did not attempt to model the processes of spore production, release and dispersal, which are governed by wind, rain and sunlight, and therefore our results should only be interpreted as relative estimates of infection risk. We did not model host phenology and assumed a constant availability of leaves over time.

Other potential weaknesses of our approach include local evolutionary adaptation, as observed in *Z. tritici* [44], which would widen the climatic niche of the species as a whole. An extreme example is the failure of a species distribution model to predict the range expansion of the Colorado Potato Beetle *Leptinotarsa decemlineata* into China, due to the evolution of burrowing behaviour that was not considered in the model [3]. Though some comparison of microclimate responses among *P. fijiensis* populations has been made (see electronic supplementary material), we have insufficient information on adaptation to adjust our model accordingly. Our modelling used the JRA55 climate reanalysis dataset, which may be subject to biases, particularly in the hydrological cycle [29]. The JRA55 model is among the most sophisticated and data-rich climate reanalyses, employing four-dimensional variational assimilation and extensive bias correction. Comparison with other reanalyses and observational data has shown JRA55 to be among the most accurate and unbiased datasets available [45]. Additionally, JRA55 is the only four-dimensional reanalysis that explicitly estimates biologically relevant parameters such as canopy temperature and canopy surface water, enhancing its utility for modelling plant pathogen epidemiology.

We employed a mechanistic model (i.e. based on mathematical abstractions of biological processes) driven by high temporal resolution (but relatively coarse spatial resolution) historical weather estimates. An alternative would have been a statistical model that correlated historical disease incidences with weather. Such observational data are not, to our knowledge, available, although we attempted to model the disease latent period in this way (see electronic supplementary material). Hence, our reliance on experimental data to parametrize the infection process in relation to temperature. Both process-based and statistical models (and models combining elements of both approaches) are widely used to model climatic effects on species distributions, biological invasions and the epidemiology and occurrence of human, animal and plant pathogens [37,39,46]. Each approach has strengths and weaknesses, and choice of a method will depend upon the data sources available, the degree of knowledge of the biological processes involved and the specific aims of the study. Statistical models are advantageous in requiring little knowledge of the biology of the system, but this simplicity makes them vulnerable to spurious extrapolations outside the range of the parametrizing data. Mechanistic models attempt to capture causality in a system and so may be more appropriate for extrapolation but can be harder to parametrize and may be highly complex for systems with many interacting processes.

Correlative models have been far more prevalent for modelling of human infectious diseases in relation to climate change [46]. For example, a linear model using growing degree days, mean saturation deficit, cumulative precipitation and distance to the ocean has been used to project future changes in Lyme disease outbreaks in the USA [47]. Application of mechanistic models to climate change effects on human disease remains relatively uncommon, perhaps because of the lack of sufficient calibration data, with only certain vector-borne and faecal-oral transmission diseases having been sufficiently studied to enable model development and parametrization [46]. The sensitivity of arthropods to weather may make vector-borne diseases particularly responsive to climate change [48].

A vector-borne human disease with a long history of mechanistic modelling is malaria [49]. The importance of determining thermal response functions for the numerous vector and parasite life cycle parameters is a key challenge to improving understanding of climate change impacts on malaria distribution, but until recently, all models used monotonically increasing functions of temperature, or ignored temperature completely [49]. Mordecai *et al.* [50] published the first unimodal response function for the basic reproductive number of malaria, determined by minimum, optimum and maximum temperatures. Unlike our beta function for *P. fijiensis* infection, quadratic and Briére functions were fitted to data from laboratory studies. The beta function was introduced for crop physiology in 1995 [51] and crop disease in 2005 [38], so it is surprising that nonlinear temperature responses have only recently been introduced into malaria modelling. As in our Black Sigatoka model, the malaria model was driven using climate reanalysis data [50], but at coarser spatial and temporal resolution reflecting the very different infection dynamics of the two diseases. Model validation was possible using a publicly available observational dataset on rates of biting by infectious mosquitoes. Such observational datasets are lacking for many plant diseases, particularly in the developing world. On the other hand, the malaria life cycle is more complex than our fungal disease, and vector-borne disease models require consideration of factors such as vector and host behaviour, habitat–weather interactions (e.g. how precipitation creates breeding areas), host immunity and socioeconomic effects (e.g. control measures) [48,49].

We did not attempt to model the potential effects of future climate on Black Sigatoka distribution and impact. Based upon temperature ranges (25–28°C) and RH (greater than 90%), declines in the area favourable to the pathogen globally and within Latin America and the Caribbean by 2080 have been projected using earlier climate projections [52]. A potential corollary of deteriorating conditions for Black Sigatoka is that growth of the host plant may also be affected. Bananas grow best in warm, moist conditions favoured by the pathogen [53]. Future drying may reduce disease risk but will also require investment in irrigation systems that could put pressure on freshwater resources [53]. Thus, the impact of future climate change on banana production from the perspective of management must consider both the disease and the host.

## Supplementary Material

Supplementary Material

## Supplementary Material

Marin.csv

## Supplementary Material

Uchoa.csv

## Supplementary Material

Guapiles.csv

## Supplementary Material

CReg.csv

## Data accessibility

JRA55 data are available from the Research Data Archive of the National Center for Atmospheric Research at https://rda.ucar.edu. SPAM crop distribution data area available from http://mapspam.info.
