# Supplementary material for: Climate change effects on Black Sigatoka disease of banana
Source: Philos Trans R Soc Lond B Biol Sci. 2019 May 6;374(1775):20180269. doi: 10.1098/rstb.2018.0269 (PMC6553611; doi:10.1098/rstb.2018.0269)
Supplement: Supplementary Material [file rstb20180269supp1.pdf]

Electronic Supplementary Material

Climate change effects on Black Sigatoka disease of banana

Daniel P. Bebber
d.bebber@exeter.ac.uk

15/03/2019

Introduction

Black Sigatoka, also known as Black Leaf Streak Disease, is a foliar disease of banana (*Musa* spp.) caused by the Ascomycete fungus *Pseudocercospora fijiensis* (previously *Mycosphaerella fijiensis*). Like many foliar fungal pathogens (Magarey et al. 2005), *P. fijiensis* requires a wet leaf surface or very high relative humidity (RH) to germinate and infect the leaf (Jacome and Schuh 1992). The rate of infection during wet or humid periods is governed by temperature (Uchôa et al. 2012).

The following discussion elaborates on the models and data we used to estimate infection risk, and presents a supplementary analysis of the latent period (the time period between infection and spore release) in relation to weather. Examples of computer code are in the R language (http://cran.r-project.org). This document was produced using the R Markdown authoring system for the RStudio interface to R (https://rmarkdown.rstudio.com).

Readers wishing to follow our coding examples should download the following files to a folder, and set their R working directory appropriately: `Uchoa.csv`, `CReg.csv`, `Marin.csv` and `Guapiles.csv`.

Infection risk model

Infection as a survival process

We treated the spore germination and infection process as a probabilistic survival process which proceeds during wet periods and has a hazard function dependent upon temperature (Bebber et al. 2016). Survival models are appropriate for infection because spores transition between states (from ungerminated spore, to germinated spore, to hypha that has penetrated a leaf). The fraction of a cohort of spores that has infected a leaf (or, equivalently, the probability that a single spore has infected a leaf) by time t is:

$$F(t) = 1 - e^{-H(t)}$$

where $H(t)$ is the cumulative hazard function. We employed a Weibull survival model, where $H(t)$ is determined by the scale α and shape γ parameters:

$$H(t) = \left(\frac{t}{\alpha}\right)^{\gamma}$$

We scaled the hazard function (and cumulative hazard) by a relative rate $r(T)$ dependent on temperature T:

$$H(t,T) = r(T)\left(\frac{t}{\alpha}\right)^{\gamma}$$

The temperature-dependant rate is a beta function determined by the cardinal temperatures (minimum, optimum and maximum) for the process:

$$r(T) = \left(\frac{T_{\max} - T}{T_{\max} - T_{\text{opt}}}\right)\left(\frac{T - T_{\min}}{T_{\text{opt}} - T_{\min}}\right)^{\frac{T\text{opt} - T\min}{T\max - T\text{opt}}}$$

so that $H(t,T)$ is greatest when $r(T) = 1$. Thus, given a temperature and a time (in hours) following the start of a wet period, we can estimate the infection intensity using a piece-wise hazard function:

$$H(t) = \sum_{i=1}^{t} r_i\left(\frac{i}{\alpha}\right)^{\gamma} - r_i\left(\frac{i-1}{\alpha}\right)^{\gamma}$$

where r_i is calculated from the mean of temperatures at the beginning and end of each hour $(T_i + T_{i-1})/2$.

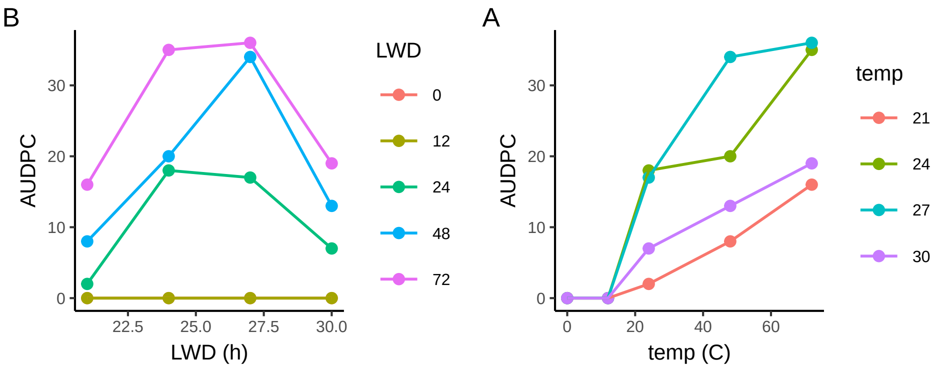

Figure 1: AUDPC by A) temperature and B) LWD, data abstracted from Uchôa (2010)

Experimental data

Uchôa et al. (2012) measured infection and disease development rates in relation to temperature and leaf wetness duration (Fig. 1). Banana plants were wetted, inoculated with conidia, and kept in plastic bags (to maintain leaf wetness) for leaf wetness durations (LWD) of 0, 12, 24, 48 or 72 hours, and at constant temperatures of 21, 24, 27 or 30 °C. The plastic bags were then removed, and plants maintained at around 55 % RH at their respective temperatures. Disease development (fraction of area affected) was monitored every four days to 24 days post inoculation, and the Area Under Disease Progress Curve (AUDPC) calculated.

We abstracted AUDPC data from a figure in the original PhD thesis (Uchôa, 2010) to parameterize a new disease development model. The use of AUDPC to investigate the combined effects of temperature and LWD on disease severity is somewhat problematic as it confounds at least two separate processes. Firstly those that take place on the leaf surface (germination, germ tube elongation, and penetration), and secondly growth within the leaf and development of visible symptoms on the leaf. Given that the Uchôa et al. (2012) experiments reduced RH to around 55 % after controlled periods of leaf wetness, we assumed that all germination and penetration occurred during the experimental wet periods, with subsequent disease development controlled solely by temperature. As we were unable to differentiate the effect of temperature on external versus internal processes, we interpreted the AUDPC as an outcome of infection driven by the germination and penetration of spores, the rates of which were dependent upon temperature.

```r
# Begin analysis
# Load required packages
library(ggplot2)
library(R.utils)
library(accelerometry)
library(plyr)
library(lubridate)
library(raster)
library(maps)
library(gridExtra)
library(viridis)
library(RColorBrewer)
library(zoo)
library(nlme)

# Load Uchôa et al. data
AUDPC <- read.csv("Uchoa.csv")
```

Model parameterization

We parameterized the model by minimizing the least squares difference between model predictions and the Uchôa data, by optimization (Fig. 2). We employed simulated annealing optimization as this method approximates the global optimum (Kirkpatrick et al. 1983).

```r
# Temperature dependent rate function
# (taking Temperature and p = c(tmin, topt, tmax))
rt <- function(Temp, p) {
  r <- ((p[3] - Temp) / (p[3] - p[2])) * ((Temp - p[1]) / (p[2] - p[1])) ^
  ((p[2] - p[1]) / (p[3] - p[2]))
  r[r < 0] <- 0 # set negative values to zero
  r[is.na(r)] <- 0 # set undefined values to zero
  r
}

# Ft with temperature-dependent rate function
# First argument is vector of parameters
Ft <- function(hr, Temp, p) {
# p  = c(scl, shp, tmin, topt, tmax, b) where b is scaling factor
# Temperature-dependent rate
r <- rt(Temp, p[3:5])
# Estimated H(t, T)
Ht <- r * (hr / p[1]) ^ p[2]
# F(t, T)
(1 - exp(-Ht))*p[6]
}

# Function to optimize by minimization of least squares
Ft_opt <- function(p, hr, Temp, obs){
  est <- Ft(hr = hr, p = p, Temp = Temp)
  sum((obs - est)^2)
}

# Estimate starting parameter values for the optimization
p <- c(30, 3, 10, 26, 40, 40)

# Simulated annealing
p_opt <- optim(par = p, fn = Ft_opt, hr = AUDPC$LWD,
               Temp = AUDPC$Temp, obs = AUDPC$AUDPC, method = "SANN",
               control = list(maxit = 10^6, temp = 20))
p_opt <- p_opt$par
names(p_opt) <- c("alpha", "gamma", "tmin", "topt", "tmax", "beta")
p_opt
```

```
##     alpha     gamma      tmin      topt      tmax      beta
## 33.080925  1.754555 16.281097 27.286143 30.263325 37.479749
```

Additional data sources

Jacome and Schuh (1992) provide further estimates for AUDPC by temperature and LWD (Fig. 3). However, they utilized fewer LWD values than Uchôa et al. (0, 9, 18 h) and a slightly different set of temperatures (22, 25, 28, 31 °C). Their experiments involved conidia of two *P. fijiensis* isolates from Honduras, denoted Santa Barbara (SB) and La Fragua (FR). As with Uchôa et al. (2012), Jacome and Schuh (1992) fitted a polynomial model to the data, which is not biologically appropriate as it assumes a symmetrical rate curve with temperature. The maximum LWD duration used by Jacome and Schuh (1992) was far shorter than that employed by Uchôa et al., and there appear to be certain anomalies such as a decline in mean AUDPC between 9 and 18 hours at 28 °C for the FR isolate. Hence, we will

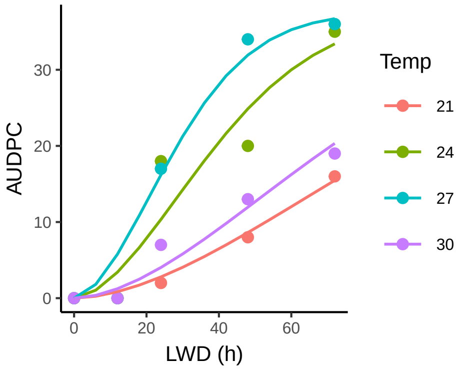

Figure 2: Optimized AUDPC curves.

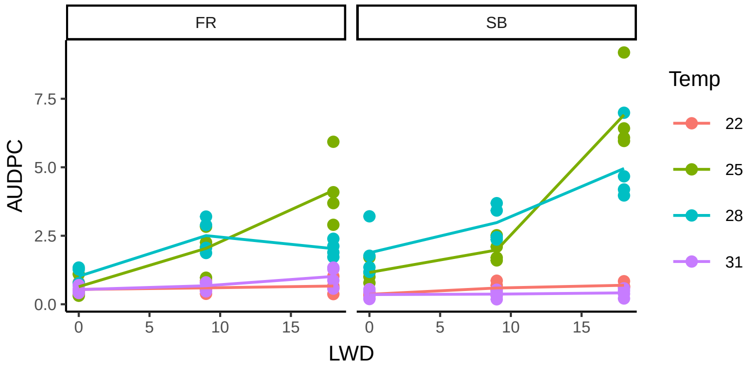

Figure 3: AUDPC by LWD and temperature, for La Fragua (FR) and Santa Barbara (SB) isolates, Honduras. Data from Jacome and Schuh (1992)

not consider this dataset further. Nevertheless, the broad pattern of increasing AUDPC with time, and cardinal temperatures of 16.7, 27.2 and 30.3 °C seem reasonable.

Microclimate data

We obtained JRA55 climate reanalysis data from the National Center for Atmospheric Research (NCAR) Research Data Archive (https://rda.ucar.edu/). The spatial extent of the dataset was tropical Latin America and the Caribbean (i.e. from 23 °S to °23 N). The temporal extent was from 00:00h UTC on 1/1/1958 to 21:00h UTC on 31/12/2017 at 3-hourly intervals. We downloaded three variables from JRA55: canopy temperature (CT), canopy surface moisture (CM), and relative humidity (RH) at 2m above ground level (Fig. 4). We analysed all land surface pixels over all hours.

Example of model results

We modelled the infection process for hypothetical cohorts of spores on an hourly basis. We did not differentiate between ascospores and conidia, assuming that the data obtained by Uchôa (2010) for conidia apply also to ascospores. In our model a cohort of spores germinates and infects during wet periods as determined by our temperature-dependent survival function, and ceases germinating and infecting once the canopy dries. A new cohort begins infecting at the beginning of each hour of a wet period. At the end of each wet period, we summed the relative number of infections for each cohort within that period.

Earlier work (Jacome et al., 1991; Jacome & Schuh, 1992) demonstrated that ascospore and conidial germination require free water on the leaf surface or RH $\geq$ 98 % Ascospore germ tube growth requires free water on the leaf surface and ceases below 98 % RH. Conidial germ tube development ceases below 92 %. Both germination and germ tube elongation rates increase roughly linearly with RH, reaching a maximum rate in liquid water. We assumed that the rate of germ tube elongation is linearly related to the probability of a germ tube entering the leaf via a stomatal pore, thereby infecting the leaf. For

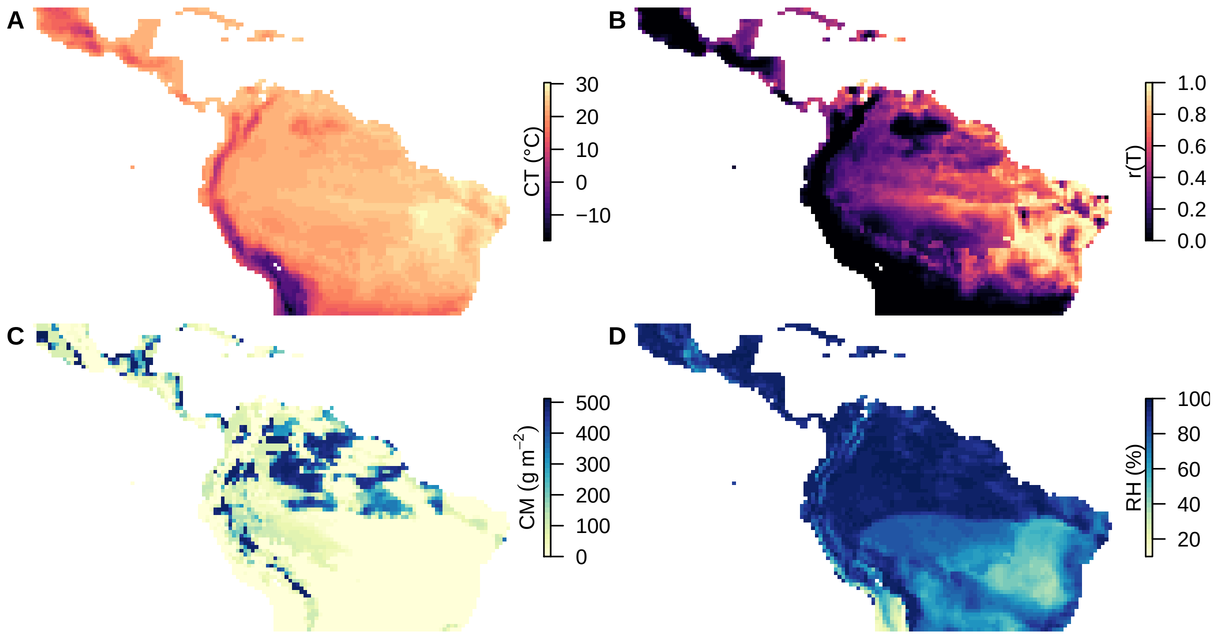

Figure 4: Example of JRA55 microclimate variables at 12:00 UTC on 1/6/2000. A) Canopy temperature (CT), B) Temperature-dependent infection rate r(T), C) Canopy surface moisture (CM), D) Relative humidity (RH) at 2m.

simplicity, we assumed that the growth (and infection) rate was governed solely by temperature, and that the presence of sufficient moisture allows the infection process to proceed (i.e. we did not adjust the rate linearly by RH).

Processing of the entire dataset is computationally intensive, so we illustrate the method using data for a pixel in Costa Rica (centered on 83.9 °W, 10.3 °N), for the month of January 2000 (Fig. 5).

```
# Load data
CReg <- read.csv("CReg.csv")
# (Time is hours after midnight on 1/1/1800, the JRA55 reference time)

# Linear interpolation function, converts 3-hourly to hourly timeseries
ins <- function(x, nas = 2){
  m <- matrix(c(x, rep(NA, length(x)*nas)), nc = length(x), byrow = TRUE)
  res <- (as.vector(m))
  res <- res[1:(length(x)*3-nas)]
  if(all(is.na(res))) return(res) else na.approx(res)
}

# Cumulative Distribution Function with temperature-dependent rate
Ftr <- function(ct, p_opt){ # takes canopy temperatures during a wet period as input
  w <- length(ct) - 1 # LWD
  ti <- matrix(ct[1:w] + diff(ct)/2, nc = w, nr = w, byrow = F) # mean temperatures
  ri <- rt(ti, p_opt[3:5]) # relative rates
  im <- matrix(1:w, nc = w, nr =  w) - rep((1:w) - 1, each= w) # end times per cohort
  im0 <- im-1 # start times
  a <- p_opt[1]; g <- p_opt[2] # Weibull survival parameters
  Hm <- ri*((im/a)^g - (im0/a)^g) # matrix giving H for each cohort and interval
  Hm[im0 < 0] <- 0 # replace values with zeros when t < 0 per cohort
  Hmc <- apply(Hm, 2, cumsum) # cumulative hazard across intervals
  Fm <- 1-exp(-Hmc) # cumulative probability matrix by cohort
  rowSums(Fm)[w] # total relative number of infecting cohorts
}
```

```r
# Function to create matrix of wet periods from vector of canopy wetness
wet.per <- function(wet){
  x <- wet > 0
  m <- rle2(as.numeric(x), indices = TRUE) # rle2 function for run lengths
  m <- matrix(m[m[,1] == 1,], nc = 4)
  m
}

# How long are wet periods during this timeseries?
rh1 <- ins(CReg$RH); cm1 <- ins(CReg$CM) # convert to hourly timeseries
ct1 <- ins(CReg$CT-273)
wet <- cm1 > 0 | rh1 >= 98 # classify each hour as wet or dry
wet.per(wet)[,4] # display only the durations
```

```
##  [1] 18 20 20 14 68 41 17 14 92 89 14 47 17 17 14 41 17 14 17 14  3
```

```r
# Function to calculate infections from:
# CM = canopy moisture 3 hr intervals, RH =  relative humidty 3 hr intervals,
# CT = canopy temperature 3 hr intervals, p_opt = vector of optimized parameters,
# Returns total infections for timeseries
infect <- function(CM, RH, CT, p_opt){ # uses 3-hourly estimates
  if(all(is.na(CT))) return(NA) else
  CT <- if(all(CT < 100)) CT else CT - 273 # convert from Kelvin if required
  cm <- ins(CM); rh <- ins(RH); ct <- ins(CT) # convert to hourly timeseries
  wet <- cm > 0 | rh >= 98 # classify each hour as wet or dry
  if(all(wet == FALSE)) return (0) else # stop calculations if always dry
  wetper <- wet.per(wet) # identify wet and dry hours
  wetper <- wetper[wetper[,4] > 3,] # keep only wet periods above 3 hours duration
  wetper <- matrix(wetper, nc = 4) # reformat
  if(nrow(wetper) == 0) return(0) else # stop if no long wet periods
  res <- list() # list for results
  for(i in 1:nrow(wetper)){ # for each wet period...
    cti <- ct[wetper[i,2]:wetper[i,3]] # take canopy temperatures for that period
    Fti <- Ftr(cti, p_opt) # calculate infections for that period
    res[[i]] <- Fti # add result to list
  }
  unlist(res) # vector of the total infections per wet period
}

# Run with example data
infect.eg <- infect(CReg$CM, CReg$RH, CReg$CT, p_opt)
round(infect.eg, 2)
```

```
##  [1]  0.28  0.44  0.33  0.04  5.65  1.42  0.08  0.07 11.63  3.77  0.02
## [12]  1.80  0.14  0.27  0.12  0.72  0.06  0.05  0.11  0.03
```

During our example period, there were 21 periods of canopy wetness, 20 of which had durations longer than 3 hours. The number of infections was greatest in the 9th wet period, which was also the longest (92 h). The 10th wet period was slightly shorter (89 h) but accumulated fewer infections, because less optimal temperatures were experienced (mean $r(T)$ 0.079 in the 9th wet period vs 0.028 in the 10th).

Geographical distribution of banana production

We used a global dataset of estimated spatial distribution of banana production to report on changes in infection risk for the major banana-producing countries across Latin America and the Caribbean. The SPAM dataset (Spatial Production Allocation Model, http://mapspam.info) provides estimates of the growing areas of 42 crops at 5 arc-minute (approx. 10 km) spatial resolution. This is a finer resolution than our climate reanalysis data (JRA55 at approx. 55 km spatial resolution) so we resampled SPAM

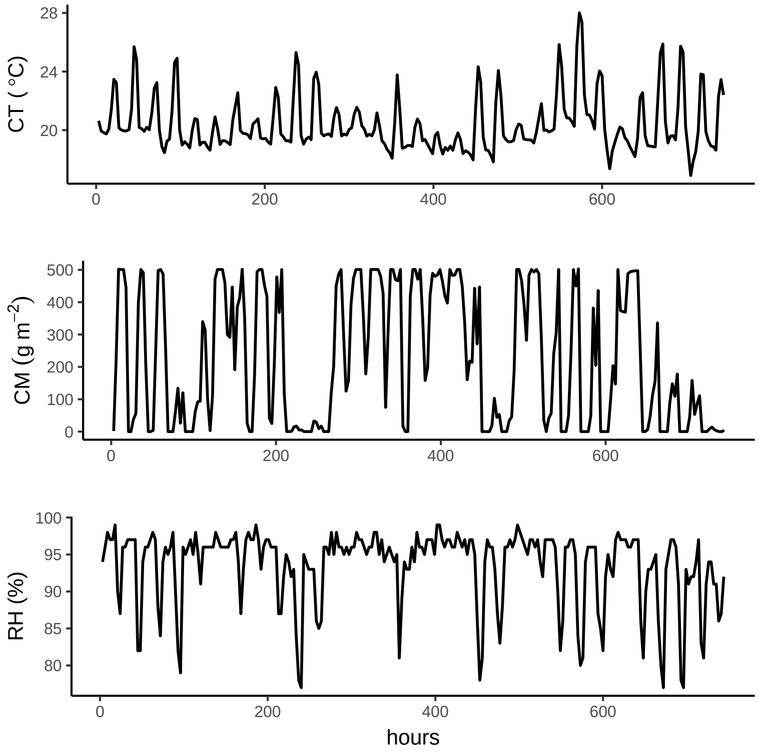

Figure 5: JRA55 microclimate by hour, January 2000 in one pixel in Costa Rica.

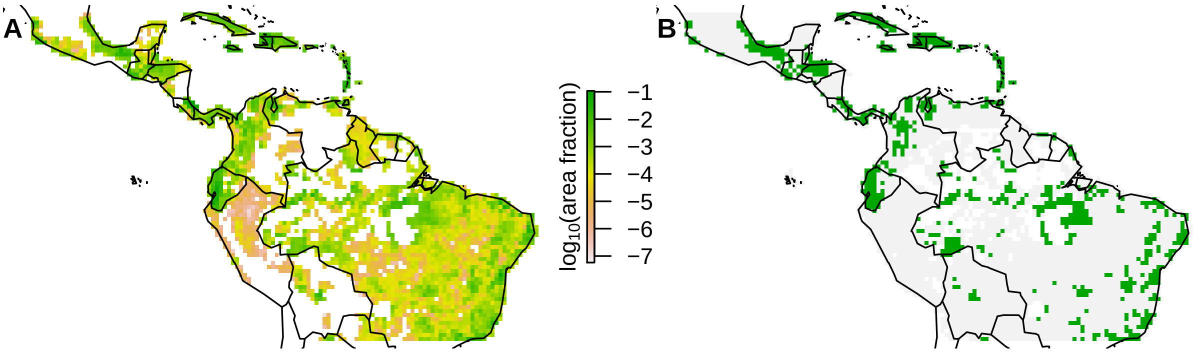

Figure 6: LAC banana production area from SPAM data. A) Fraction of JRA55 pixel, B) JRA55 pixels with > 0.1 % cover.

data to the coarser resolution to allow selection of banana-growing areas for further analysis. Areas with relatively high (> 0.1 %) banana cultivation are scattered throughout the region, and we used these areas in calculations of trends in infection risk for the most important banana-producing countries (Fig. 6).

Sub-pixel climatic variation

JRA55 reanalyses are provided at 0.5625° × 0.5625° (9/16th degree) spatial resolution, equating to 59.9 - 62.4 km squares over the latitudinal range of the regions of interest. Within each grid square, significant variation in local microclimate is likely. The estimates provided by JRA55 represent model projections for mean conditions per pixel. Land surface temperature estimates takes mean land surface altitude into account, reflected in the cooler temperatures observed over the Andes and other mountain ranges. In Latin America and the Caribbean, Cavendish bananas tend to be grown in low elevation areas, which are warmer and thus more productive. The elevational lapse rate of temperature is around -6.5 K/km (ICAO 1993), therefore we must consider adjusting JRA55 temperatures upwards if pixels contain high altitude areas.

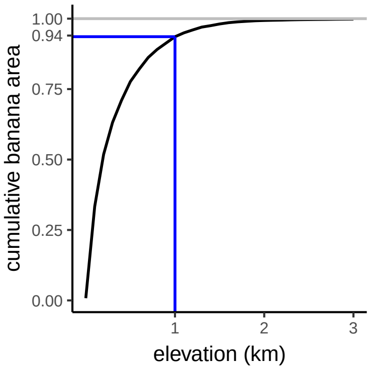

Figure 7: Cumulative area fraction under banana cultivation by altitude for the region of interest, from SPAM data.

We used the Global 30 Arc-Second Elevation (GTOPO30) dataset (available from https://lta.cr.usgs.gov/GTOPO30) to estimate temperature variability within pixels due to the lapse rate. We resampled both GTOPO30 and SPAM banana area to 1/16°, giving 9 × 9 subpixels per JRA55 pixel. This allowed us to determine the altitudinal distribution of banana area by altitude, showing that 94 % of banana growing area occurs below 1000 m elevation (Fig. 7). For each JRA55 pixel with > 0.1 % banana growing area, we calculated the deviation of subpixel altitudes from the mean, converted this to a temperature differential using the lapse rate, and recalculated the mean temperature differential from those subpixels below 1000 m elevation. We found that, of the 830 banana-growing pixels, only five had an elevation-adjusted temperature differential of > 0.5 °C, of which three were in Chile and two in Venezuela, while 750 pixels were entirely below 1000 m in elevation. Therefore, we did not adjust the JRA55 canopy temperatures for elevation when modelling infection. Estimating the within-pixel spatial variability of canopy moisture content and relative humidity would be a far more complex task, and beyond the scope of this analysis.

Black Sigatoka infection risk and range expansion

Black Sigatoka was first reported in Latin America in Honduras in 1972. The disease appears to have been introduced on infected plants imported from South-east Asia for a commercial plant breeding programme (Robert et al., 2015). The disease then spread throughout the continent and Caribbean islands, most recently reaching Martinique in 2011. We analysed the relationship between modelled infection risk and the year of first reporting of the disease in different countries (Fig. 8). There was no indication that first detection of the disease occurred at some threshold value of infection risk.

Weather Effects on Incubation and Latent Period

Our weather-driven infection risk model considers only the fraction of spores that germinate and infect leaves. Once hyphae have penetrated the leaf, they proliferate within and eventually form lesions that release ascospores which then disperse and cause further cycles of infection (Marín et al. 2003). The shorter the period between infection and spore release, the more rapidly an epidemic can develop. Hence, the factors controlling this period are key to understanding the epidemiology of BS.

Disease development data

The *incubation period* t_I is defined by the time between infection and appearance of symptoms, and in an experiment in Costa Rica t_I was found to vary between 14 and 35 days (Marín et al. 2003). The first symptom of Black Sigatoka is known as *mark*, a depigmentation on the lower surface of the leaf. The *latent period* t_L is defined as the time between infection and the formation of mature pseudothecia and ascospores, and in the same Costa Rica study t_L varied from 25 days to 70 days (Marín et al. 2003). [Note: precise quantitative definitions of incubation and latent periods vary among studies.]

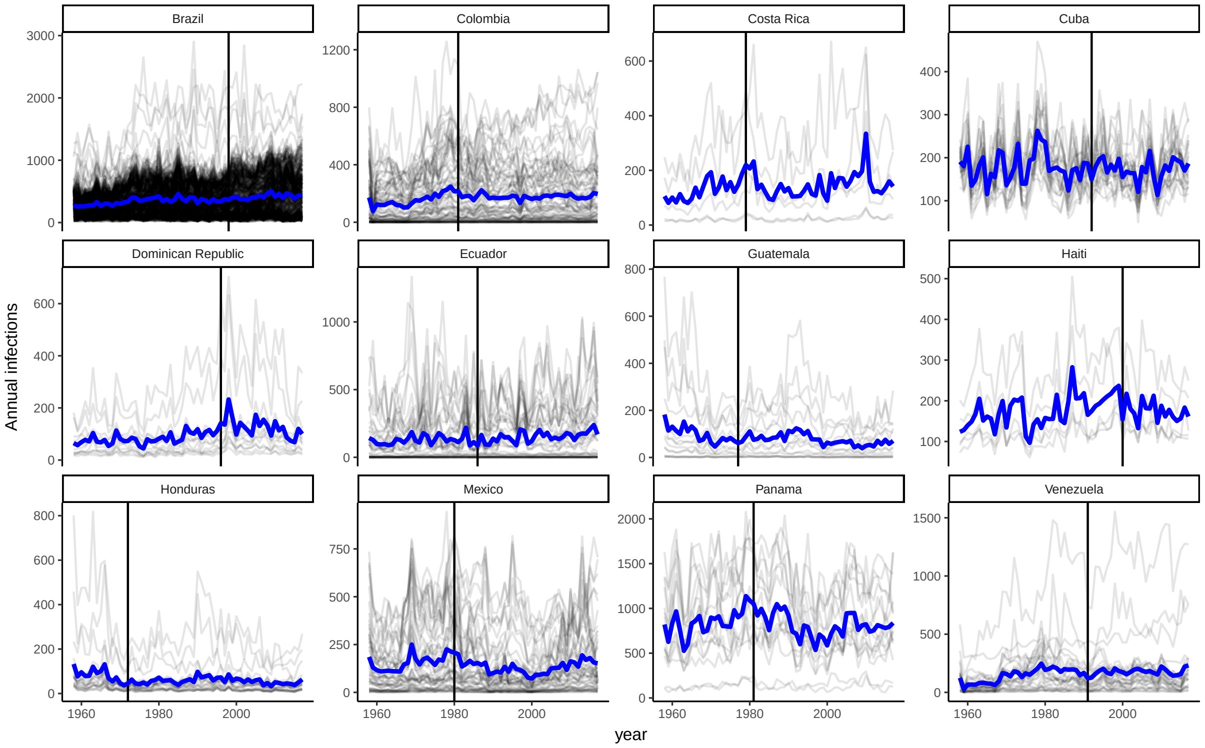

Figure 8: Modelled annual infection for the top 12 banana-producing countries, 1958-2017. Grey lines show results for individual pixels with banana area >0.1 %. Blue lines show area-weighted means. Vertical black lines show year that Black Sigatoka was first reported.

We abstracted data on t_I and t_L from figures in Marín et al. (2003), giving 51 data points for each variable, spanning the period 12/12/1993 to 02/05/1995 (Fig. 9). The only information given on the study design was that each value corresponds to a mean of 10 leaves of the Grande Naine cultivar, selected at stage 2 of the cigar leaf unfurling on the indicated date, and that the study took place in Guapiles, Costa Rica. In the original figures, the starting date of each replicate is not printed on the figure axis, hence we estimated the missing start dates from the length of the intervals between the given dates.

```
# Load Marin data on latent and incubation periods
marin <- read.csv("Marin.csv", stringsAsFactors = FALSE)
marin$Date <- as_date(marin$Date)

# How much longer is latent period than incubation period?
marin.lm <- lm(Latent ~ Incubation, marin)
confint(marin.lm)
```

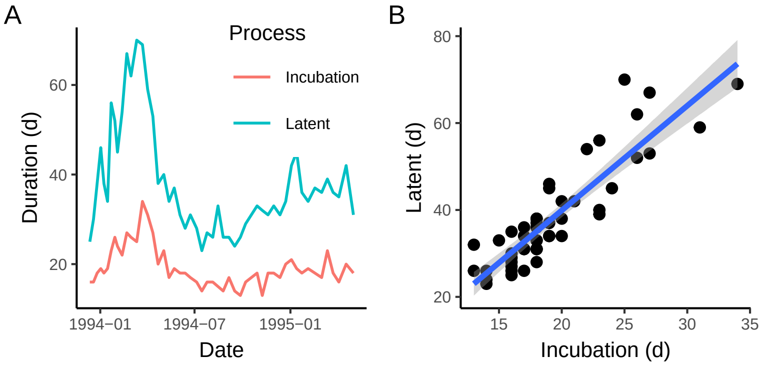

Figure 9: Incubation and latent periods, data from Marín et al. (2003)

```
##                  2.5 %    97.5 %
## (Intercept) -15.295657 -1.297260
## Incubation    2.055036  2.763944
```

The linear model indicates that t_L is 2.06 to 2.76 times longer (95 % CI) than t_I.

Weather data

Marín et al. (2003) state that favourable weather conditions minimize t_I and t_L, but the precise nature of those weather conditions are not discussed. We investigated the influence of weather on disease development with JRA55 estimates of plant canopy temperature and relative humidity from December 1993 to December 1995, the period spanning the observations of disease development in Marin et al. (2003), for a single pixel centered on 83.53125 °W, 10.40625 °N which covers the city of Guapiles.

Statistical model

We analysed the relationship between t_L and t_I, and the weather. We assumed that infection had been successful in each experimental inoculation (otherwise we would not see disease development), and so development of disease within the plant should be primarily determined by temperature. We assumed no knowledge of cardinal temperatures determining disease development rate, and simply related t_L and t_I to the average temperatures during those periods. We hypothesized that there would be an optimum temperature with the fastest disease development rate.

```r
# Load JRA55 weather data for Guapiles
guap <- read.csv("Guapiles.csv")

# Convert JRA55 time data to POSIX time and Date formats
guap$Time <- as_datetime(3600*as.numeric(guap$Time), origin = "1800-01-01")
guap$Date <- as_date(guap$Time)

# Calculate Incubation and Latent dates
marin$DateI <- marin$Date + marin$Incubation
marin$DateL <- marin$Date+ marin$Latent

# Function for mean temperature over disease development periods
meanweather <- function(dates, guap, v = "CT"){
  dates <- as.vector(dates)
  dats <- guap[guap$Date >= dates[1] & guap$Date <= dates[2],]
  mean(dats[[v]])
}

# Apply to Incubation and Latent period dates
marin$TempI <- apply(cbind(marin$Date, marin$DateI), 1, meanweather, guap, "CT")
marin$TempL <- apply(cbind(marin$Date, marin$DateL), 1, meanweather, guap, "CT")
```

There was no clear relationship between mean temperature over the disease development period, and the length of the period (Fig. 10). We therefore conducted the same analysis using relative humidity, under the hypothesis that plant water status influences disease development rate.

```r
## Effect of relative humidity on disease
# Apply to Incubation and Latent period dates
marin$RHI <- apply(cbind(marin$Date, marin$DateI), 1, meanweather, guap, v = "RH")
marin$RHL <- apply(cbind(marin$Date, marin$DateL), 1, meanweather, guap, v = "RH")
```

Disease development (for both incubation and latent periods) declined with mean relative humidity, reaching an asymptote at around 92 % RH (Fig. 11). We fitted asymptotic nonlinear regressions to the data, and compared these to a simple linear model by AIC. The analyses suggest that an asymptotic relationship fits the data better than a linear relationship. The minimum incubation was 15.0 ± 1.7 d

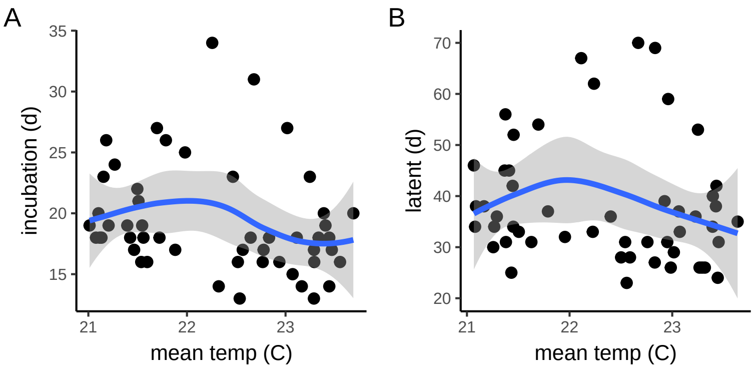

Figure 10: Disease development vs. temperature. A) Incubation period, B) Latent period.

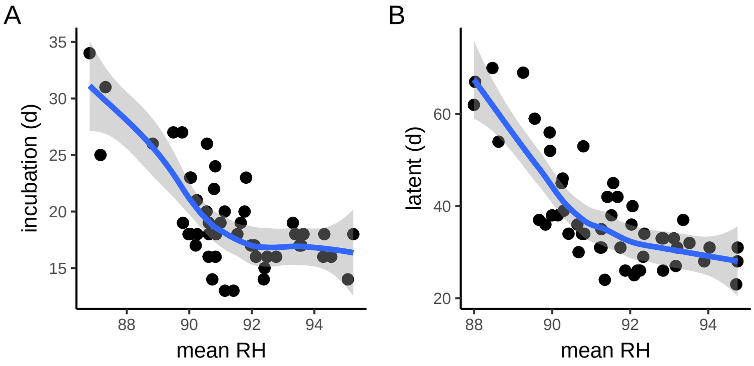

Figure 11: Disease development vs. RH. A) Incubation period, B) Latent period.

(approximate 95 % confidence interval of 12.3 – 19.4 d). The minimum latent period was 24.6 ± 4.2 d (95 % CI 17.7 – 36.8 d).

```
# Fit asymptotic models
i.nls <- nls(Incubation ~ SSasymp(RHI, Asym, R0, lrc), data = marin)
l.nls <- nls(Latent ~ SSasymp(RHL, Asym, R0, lrc), data = marin)
lt.nls <- nls(Latent ~ SSasymp(RHL, Asym, R0, lrc), data = marin)

# Fit linear models
i.lm <- lm(Incubation ~ RHI, data = marin)
l.lm <- lm(Latent ~ RHL, data = marin)

# Compare the AIC (nls gives more negative AIC = is better)
AIC(i.lm); AIC(i.nls)
```

```
## [1] 271.6339

## [1] 259.7785
```

```
AIC(l.lm); AIC(l.nls)
```

```
## [1] 361.1582

## [1] 347.2528
```

We then determined whether temperature had any influence on t_I and t_L once mean RH has been taken into account.

```
marin$RHIresid <- resid(i.nls)
marin$RHLresid <- resid(l.nls)
```

The residuals of the asymptotic fit of RH to t_I and t_L were not dependent upon temperature (Fig. 12). The ranges of values for mean temperature and RH in this analysis were very narrow, making

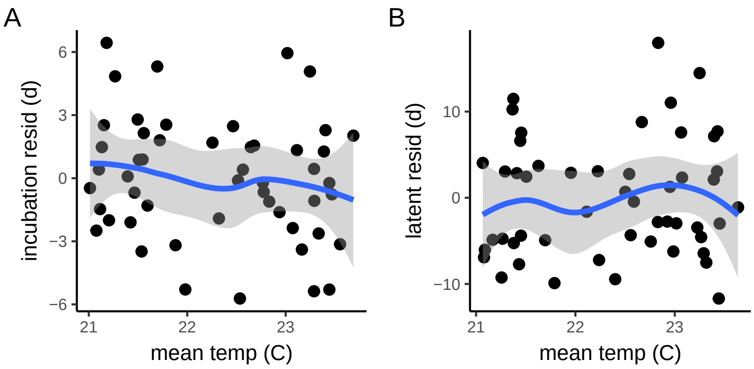

Figure 12: Residuals of asymptotic fits of disease development periods against mean RH. A) Incubation period, B) Latent period.

extrapolation difficult. Hence, we attempted no modelling of t_L across the region of interest.

The result that disease development period declines with RH is remarkable for three reasons. Firstly, RH is an atmospheric variable but disease development prior to lesion formation occurs within the leaf. Hence, it is possible that RH is affecting plant physiology in some way that enhances fungal growth within the leaf. Field data from Taiwan indicate that the rate of disease incidence change is partly determined by the RH and precipitation in the preceding month (Chuang 1987), but in that case inoculation was natural as compared to the artificial inoculation in the Guapiles experiment. Hence, further work is required to verify and determine mechanisms for an effect of RH on disease development. Secondly, the lack of a relationship with temperature is surprising because fungal pathogen growth is dependent on leaf temperature under experimental conditions (Bernard et al. 2013). One possible explanation is that the range of mean canopy temperatures during disease development was too small to give detectable differences in t_L. However, our disease development rate model suggests that at the lowest mean canopy temperature in the study (21.1°C) the rate would be 0.14, while the rate at the highest mean canopy temperature (23.6 °C) the rate would be 0.50, a large difference. Another explanation could be that the JRA55 canopy temperature data are too spatially coarse for this analysis, which brings us to the third point. The detection of a strong relationship between t_L derived from an experimental study and mean RH derived from a global reanalysis of climate data at coarse spatial resolution was unexpected, and provides some confidence that JRA55 can give meaningful weather estimates at sub-pixel resolution. However, the lack of a detectable effect of temperature suggests caution, and the drawing of conclusions over wider regions where results from multiple pixels can be pooled.

References

Bebber DP, Castillo ÁD, Gurr SJ. 2016 Modelling coffee leaf rust risk in Colombia with climate reanalysis data. Phil. Trans. R. Soc. B 371, 20150458. https://dx.doi.org/10.1098/rstb.2015.0458

Bernard F, Sache I, Suffert F, Chelle M. 2013 The development of a foliar fungal pathogen does react to leaf temperature! New Phytol 198, 232–240. https://dx.doi.org/10.1111/nph.12134

Chuang TY. 1987 Predicting the Rate of Development of Black Sigatoka (Mycosphaerella fijiensis var. difformis) Disease in Southern Taiwan. Phytopathology 77, 1542. https://dx.doi.org/10.1094/Phyto-77-1542

Fischer G, Nachtergaele FO, Sylvia Prieler, Teixeira E, Tóth G, van Velthuizen H, Verelst L, Wiberg D. 2012 Global Agro-Ecological Zones (GAEZ v3.0). IIASA and FAO, 178.

ICAO 1993. Manual of the ICAO Standard Atmosphere (extended to 80 kilometres (262 500 feet)) (Third ed.). International Civil Aviation Organization.

Kirkpatrick S, Gelatt CD, Vecchi MP. 1983 Optimization by simulated annealing. Science 220:671-680. https://dx.doi.org/10.1126/science.220.4598.671

Magarey RD, Sutton TB, Thayer CL. 2005 A Simple Generic Infection Model for Foliar Fungal Plant Pathogens. Phytopathology 95, 92–100. https://dx.doi.org/10.1094/PHYTO-95-0092

Marín DH, Romero RA, Guzmán M, Sutton TB. 2003 Black Sigatoka: An Increasing Threat to Banana Cultivation. Plant Disease 87, 208–222. https://dx.doi.org/10.1094/PDIS.2003.87.3.208

Robert S, Zapater MF, Carlier J, Abadie C, Ravigne V. 2015 Multiple introductions and admixture at the origin of the continental spread of the fungal banana pathogen #Mycosphaerella fijiensis# in Central America: a statistical test using approximate bayesian computation. Revue d'Ecologie 70, 127–138.

Uchôa C do N. 2010 Estudos epidemiológicos e diagnose molecular da Sigatoka-Negra. PhD, Universidade Federal de Lavras, Lavras, Brazil.

Uchôa C do N, Pozza EA, Albuquerque KS, Moraes W da S. 2012 Relação entre a temperatura e o molhamento foliar no monocíclo da Sigatoka-negra. Summa Phytopathologica 38, 144–147. https://dx.doi.org/10.1590/S0100-54052012000200006
